# Induction of integrin α_2_ in a highly bone metastatic human prostate cancer cell line: roles of RANKL and AR under three-dimensional suspension culture

**DOI:** 10.1186/1476-4598-13-208

**Published:** 2014-09-08

**Authors:** Shabnam Ziaee, Leland WK Chung

**Affiliations:** Departments of Biomedical Sciences, Samuel Oschin Comprehensive Cancer Center, Cedars-Sinai Medical Center, Los Angeles, CA 90048 USA; Medicine, Samuel Oschin Comprehensive Cancer Center, Cedars-Sinai Medical Center, Los Angeles, CA 90048 USA; Department of Medicine and Surgery, Uro-Oncology Research, Samuel Oschin Comprehensive Cancer Center, Cedars-Sinai Medical Center, 8750 Beverly Blvd. Atrium 103, Los Angeles, CA 90048 USA

**Keywords:** 3-D culture, Androgen Receptor, AP-4, Cell Adhesion, Collagen Type I, Integrin α_2_, Prostate cancer

## Abstract

**Background:**

Prostate cancer (PCa) bone metastasis can be markedly enhanced by increased receptor activator of NF kappa-B ligand (RANKL) expression in PCa cells. Molecular mechanisms that account for the increased predilection of PCa for bone include increased bone turnover, promotion of PCa cell growth and survival in the bone environment, and recruitment of bystander dormant cells to participate in bone metastasis. The current study tests the hypothesis that PCa cells acquire high adhesion to bone matrix proteins, which controls PCa bone colonization, under the RANKL/RANK and AR axes.

**Methods:**

We used a highly bone metastatic RANKL-overexpressing LNCaP PCa cell line, LNCaP^RANKL^, as a model to pursue the molecular mechanisms underlying the increased adhesion of PCa cells to collagens. A three-dimensional (3-D) suspension PCa organoid model was developed. The functions of integrin α_2_ in cell adhesion and survival were evaluated by flow cytometry and western blot. AR expression and functionality were compared in 2-D monolayer versus 3-D suspension cultures using AR promoter- and PSA promoter-luciferase activity. AR role in cell adhesion was assessed using an adhesion assay.

**Results:**

LNCaP^RANKL^ cells were shown to adhere tightly to ColI matrix through increased α_2_ integrin expression. This increased adhesion, concomitant with activation of the FAK and Akt pathways, was further enhanced by culturing LNCaP^RANKL^ cells in 3-D suspension. Under the influence of 3-D suspension culture, AR was restored in LNCaP^RANKL^ cells via downregulation of AP-4 transcription factor, and supported increased α_2_ integrin expression and adhesion to ColI.

**Conclusion:**

3-D suspension culture and *in vivo* PCa tumor growth restore AR through downregulation of AP-4, enhancing integrin α_2_ expression and adhesion to ColI which is rich in bone matrices. The interactions of PCa with ColI, mediated by integrin α_2_ and AR expression, could be a key molecular event accounting for PCa bone metastasis.

**Electronic supplementary material:**

The online version of this article (doi:10.1186/1476-4598-13-208) contains supplementary material, which is available to authorized users.

## Introduction

Prostate cancer (PCa) has the highest incidence and is the second most common cause of cancer death among men in western countries [1]. The main clinical complication causing morbidity [2, 3] and mortality in PCa patients is bone metastasis, which presents in over 80% of all men who die of PCa [4, 5]. Despite the high occurrence of skeletal metastasis, the underlying molecular mechanisms determining the predilection of PCa cells for homing to bone are not well-understood. Previously, we hypothesized that the osteomimetic properties of PCa cells account for the predilection of PCa to metastasize and grow in the bone microenvironment [6]. We found that β-2 microglobulin (β-2 M), a major histocompatibility co-receptor, mediates the expression of non-collagenous bone matrix proteins such as osteocalcin and bone sialoprotein in metastatic human prostate cancer cell lines [7]. We found that upon the induction of β-2 M, prostate cancer cells overexpress RANKL, a protein intimately related physiologically to bone turnover [8]. RANKL drives PCa cells to undergo epithelial-to-mesenchymal transition (EMT) [9, 10], and when expressed by human cancer cell lines like LNCaP^RANKL^, produces explosive skeletal and soft tissue metastases upon intracardiac administration in mice [11].

Overexpression of RANKL plays a role in the breast cancer osteolytic phenotype by binding to its RANK receptor on precursor osteoclasts [12]. Recent studies have shown that RANKL positively correlates with higher Gleason score in PCa [13] and predicts the survival of PCa patients [14]. Denosumab, an anti-RANKL antibody approved by the FDA for the management of osteoporosis and breast and prostate cancer bone metastasis, has been shown to improve or delay skeletal metastasis in breast and prostate cancer by 35% [15] and 18% [16], respectively. However, overall patient survival is not affected, indicating the critical roles of other potential factors affected by the RANK-mediated downstream signaling network in PCa bone metastasis.

Another important factor in the development and progression of PCa is androgen receptor (AR) [17]. AR has regulatory roles promoting PCa cell adhesion and survival in bone. PCa cells are initially androgen-sensitive (AS) and respond to androgen deprivation therapy (ADT) [17]. Overtime, while PCa cells remain AR positive, they progress to become androgen-insensitive (AI) and acquire increased invasiveness and metastatic potential [18, 19]. AI tumors in hosts subjected to ADT become hypersensitive to residual intracrine androgen due in part to AR gene amplification, AR gene mutation, and/or higher AR regulating transcription factors (TFs) [20–22]. Recently, AR was found to induce cancer cell adhesion and survival through integrin expression [23–25]. Since AR plays a significant role in PCa metastasis, understanding how AR affects PCa adhesion to collagen matrix in bone could provide potential therapeutic approaches to block PCa bone homing and increase patient survival.

Multivariable tumor and microenvironmental factors are known to engage in tumor development and progression. Current 2-D monolayer culture lacks the relevant cell–cell and cell–matrix interactions that occur physiologically in the *in vivo* environment. This limitation makes it extremely difficult or potentially impossible to define the key cell signaling networks supporting essential cellular functions *in vitro* [26, 27]. Extracellular matrix (ECM) mediates biological and physical cues external to the cell that result in altered cell proliferation, migration, invasion, and adhesion. Cell-ECM communication is initiated through the interaction of α- and β-integrin subunits to specific extracellular matrices [28, 29] activating cell signaling pathways such as cell focal adhesion kinase (FAK) [30, 31]. 3-D *in vitro* models have an invaluable ability to recapitulate some of the *in vivo* cell-cell and cell-ECM interactions governing tumor cell behavior [32, 33].

In the present investigation, we used 3-D models to test the possibility that increased PCa adhesion to bone-derived ECM could promote PCa homing to bone. The objectives of this study were: 1) To investigate if RANKL overexpression promotes overexpression of integrins that support the adhesion of PCa cells to bone matrix proteins; 2) To determine if the levels of integrin expression are affected by growing PCa cells in 3-D suspension culture; 3) To determine if AR can be restored in RANKL-overexpressing LNCaP cells, and whether this restored AR modulates integrin expression/function to increase the growth, adhesion and survival of PCa cells in bone. To the best of our knowledge, we illustrated for the first time that overexpression of RANKL in human PCa cells induced dramatic upregulation of integrin α_2_ expression which facilitated the adhesion of PCa cells, specifically to collagen type I (ColI). We assessed and compared the adhesion of PCa cells to ColI in 2-D vs. 3-D culture, and determined the roles of FAK and Akt activation in PCa adhesion and survival. We further assessed the overall effects of AP-4, a newly identified regulator of AR, on cell adhesion to ColI via increased α_2_ integrin expression.

## Results

### Comparison of LNCaP^Neo^ and LNCaP^RANKL^ cell adhesion, integrated motility, and migration

**Figure 1:**
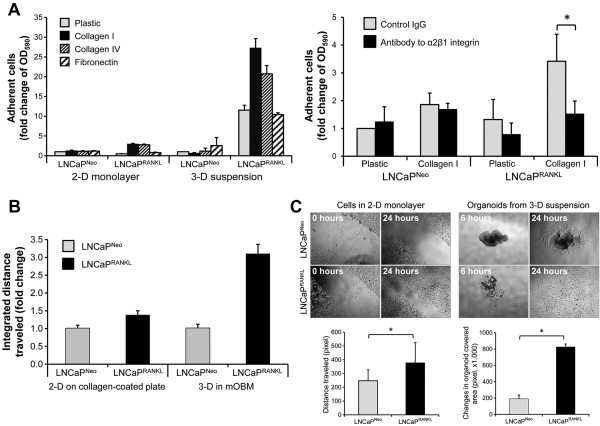
**LNCaP** ^**RANKL**^ **cell adhesion and migration are enhanced in 3-D suspension culture. (A)** In the left panel, RANKL expression is shown to enhance the adhesion of PCa cells to ColI. For each condition, 5,000 single cells from 2-D monolayer or 3-D suspension culture were seeded on 96-well plates pre-coated with ColI, ColIV or FN. After 30 minutes of incubation, the number of adherent cells was determined using alamarBlue assay. LNCaP^RANKL^ cells preferentially adhered to ColI, especially when the cells were pre-conditioned in 3-D suspension culture. In the right panel, a similar assay was conducted with cells pre-conditioned in 3-D suspension culture, where antagonizing antibody to α_2_β_1_ integrin was introduced to interfere with adhesion. The results are presented along with ratios to the control LNCaP^Neo^ cells for each condition. Each data point is the mean ± SD of 6 measurements from 2 independent experiments. **(B)** Time lapse fluorescence microscopy was used to determine cell motility. Compared to control, motility of LNCaP^RANKL^ cells was enhanced by 3 fold when the cells were pre-conditioned in 3-D suspension culture. Integrated distance traveled was normalized to LNCaP^Neo^ cells and presented as the mean ± SD of 5 separate experiments. **(C)** Pre-conditioning in 3-D suspension culture increased the migration potential of LNCaP^RANKL^ cells. In the upper left panels, cells in 2-D monolayer culture on ColI-coated plates were subjected to a wound healing assay. In the upper right panels, migration of cells pre-conditioned in 3-D suspension culture was assessed. In the lower panels, changes in cell migration were quantified based on the results of 3 separate experiments.

Previous studies established that RANKL-overexpressing LNCaP or ARCaP cells metastasized to bone and soft tissues when inoculated intracardially [11, 34]. We used the RANKL-transfected LNCaP cell line, LNCaP^RANKL^, to test the possibility that increased PCa cell homing to mouse skeleton could be due to increased cell adhesion and migration through a rise in integrin expression. We determined differential adhesion, integrated motility, and migration between LNCaP^Neo^ and LNCaP^RANKL^ cells under 2-D versus 3-D growth conditions. Prior to the use of 3-D conditions, we extensively compared the pros and cons of culturing PCa cells under 2-D versus 3-D using different substrata consisting of Matrigel, Hydrogel, polymeric PLGA mesh, and suspension culture in the presence or absence of ColI. The morphologic features of PCa cells under 2-D and 3-D growth conditions and their pros and cons are presented in Additional file 1: Figure S1 and Additional file 2: Table S1. Based on these comparative studies, we concluded that 3-D suspension culture has the definitive advantages of simplicity, ease of expanding into large scale culture, low cost, and production of spheroid structures that can be easily handled for histopathologic and immunohistochemical analyses of the cultured cells. After these comparative studies, we compared the adhesion and migration of LNCaP^Neo^ and LNCaP^RANKL^ cells cultured in a 2-D monolayer rather than 3-D suspension. Figure 1A shows that LNCaP^RANKL^ cells attached to the ColI and collagen IV (ColIV) extracellular matrices, better than LNCaP^Neo^ cells. Results indicate that the higher adhesion of LNCaP^RANKL^ cells to ColI-coated plates was further enhanced when they were pre-grown in 3-D suspension culture (Figure 1A; left panel). As expected, the increased adhesion of LNCaP^RANKL^ cells to ColI can be antagonized by an anti-α_2_β_1_ antibody, where a 55% reduction of cell adhesion to ColI was observed within 30 min (Figure 1A; right panel). We noted that LNCaP^RANKL^ cells anchored to ColI much more rapidly (within 30 minutes) under 3-D suspension compared to growth in 2-D monolayer or compared to LNCaP^Neo^ cells. The adhesion difference between the two cell lines and among different ECMs was not significant after 3 hours (Additional file 3: Figure S2). As illustrated, LNCaP^RANKL^ cells also exhibited higher adhesive properties to ColIV but not to FN or plastic under suspension culture conditions. In sharp contrast, LNCaP^Neo^ cells had no preferential binding to collagens (I and IV) under any of the culture conditions tested. The higher ColIV binding preference of LNCaP^RANKL^ cells could explain their higher invasiveness through basement membranes compared to their parental control LNCaP^Neo^ cells, as described previously [11]. In support of this observation, LNCaP^RANKL^ cells exhibited greater integrated cell motility in 3-D mOBM containing ColI [35] than on 2-D ColI-coated plates, when compared to LNCaP^Neo^ cells (Figure 1B). We also found that while LNCaP^RANKL^ and its parental cell line have the same growth rate (data not shown), LNCaP^RANKL^ cells migrate farther than LNCaP^Neo^ cells under 3-D suspension conditions and in the presence of ColI (Figure 1C).

### Increased integrin α_2_ mediates activated phosphorylation of FAK and Akt in LNCaP^RANKL^ cells under 3-D suspension growth

**Figure 2:**
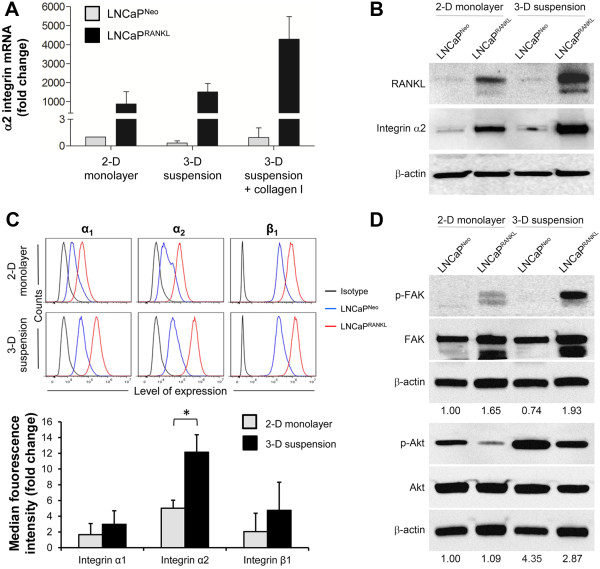
**RANKL overexpression induces integrin α**_**2**_ **expression, which mediates FAK and Akt phosphorylation. (A)** LNCaP^RANKL^ cells expressed increased α_2_ integrin as determined with qRT-PCR. The expression was the highest when the cells were grown in the 3-D suspension that contained ColI. **(B)** Increased α_2_ integrin expression, appearing in a RANKL-dependent manner, was confirmed with western blotting. **(C)** In the upper panels, cell surface expression of α_1_, α_2_ and β_1_ integrins was detected with FACS. Results indicate significantly higher α_2_ integrin expression in RANKL-overexpressing cells grown in 3-D suspension. In the lower panel, the histogram represents the ratio of the surface integrin protein level of LNCaP^RANKL^/LNCaP^Neo^ cells quantified as median fluorescence intensity. Each value is the mean ± SD of two independent experiments. **(D)** Increased α_2_ integrin is accompanied by higher p-FAK and p-Akt levels as determined by western blotting. For each group, the ratio of p-FAK/FAk and p-Akt/Akt normalized to LNCaP^Neo^ is shown. Blots were cropped to emphasize the relevant bands.

Among the known receptors for ColI, α_2_β_1_ subunits are shown to be specific to ColI and play a critical role in PCa [36, 37]. Our preliminary comparative microarray analysis of 2-D monolayer grown LNCaP^Neo^ and LNCaP^RANKL^ cells revealed increased α_2_ integrin expression (Additional file 4: Figure S3A). Using microarray data, we also found that integrin α_2_ expression in LNCaP^RANKL^ cells was further enhanced by subjecting LNCaP^RANKL^ cells to 3-D suspension culture (Additional file 4: Figure S3A). mRNA and protein expression of integrin α_2_ was performed in each condition and confirmed the microarray data (Figure 2A, 2B). qRT-PCR analysis of cell embedded in suspension containing 0.1 mg/ml ColI suggests that higher expression of integrin α_2_ in cells could be further triggered in the presence of ColI (Figure 2A). These results were confirmed by FACS analysis comparing integrin α_2_ expression between LNCaP^Neo^ and LNCaP^RANKL^ cells cultured under 2-D monolayer and 3-D suspension conditions (Figure 2C). Quantitative analysis of FACS data revealed that integrin α_2_expression of LNCaP^RANKL^/LNCaP^Neo^ cells increased by 2.4 fold when cells were cultured in 3-D suspension as opposed to 2-D monolayer culture. FACS analysis did not show any significant changes in α_1_ and β_1_ integrin expression. Increase in integrin α_2_ expression appeared to be controlled by the RANKL/RANK axis, as the protein expression of RANKL correlates with integrin α_2_ expression (Figure 2B). This was further confirmed by using LNCaP^RANKL^ cells with RANK knocked down. Disrupting the RANKL/RANK pathway resulted in reduced mRNA and protein expression of integrin α_2_ (Additional file 4: Figure S3B). Interestingly, the protein expression of RANKL of LNCaP^RANKL^ cells grown in the 3-D suspension culture illustrates expression of the smaller band besides the total RANKL. This band could represent a soluble RANKL. In a parallel study using Elisa assay we have shown that soluble RANKL only increases by 7% in LNCaP^Neo^ cells when compared 3-D suspension with 2-D monolayer. However, this difference increases to 30% in LNCaP^RANKL^ cells. Higher soluble RANKL in 3-D suspension could be explained by potentially higher MMP7 expression in this condition, which is known to be responsible for the cleavage of RANKL [38]. Corresponding with the increased integrin α_2_, we also observed that LNCaP^RANKL^ cells expressed higher levels of phosphorylated focal adhesion kinase (FAK) and phosphorylated Akt, when compared to 2-D monolayer (Figure 2D). Interestingly, under 3-D suspension conditions, LNCaP^Neo^ parental cells showed slightly lower p-FAK expression, while Akt phosphorylation was significantly higher. These data in aggregate suggest that RANKL-expressing PCa cells grown in 3-D suspension have elevated cell adhesion and survival capability and this is likely mediated by activated α_2_β_1_ integrin.

Other ECM receptors have been shown to play a role in PCa invasion and migration including ColIV receptor, α_1_β_1_ [39], laminin receptors, α_3_β_1_ [40] and α_6_β_1_ [25], and fibronectin receptor, α_v_β_3_ [41]. We compared the microarray expression of α_1_, α_3_, α_6_, α_v_, β_1,_ and β_3_ between LNCaP^Neo^ and LNCaP^RANKL^ cells. Other than increased integrin α_2_ expression, only integrin α_v_β_3_ showed significantly increased expression in 3-D suspension versus 2-D monolayer culture (Additional file 4: Figure S3A). However, we could not confirm the differential expression of this integrin by FACS analysis (data not shown). Additionally, we analyzed the cell surface expression of integrin α_2_ in the androgen-refractory PCa cancer cell line, ARCaP. Upon malignant progression, ARCaP_M_ cells are known to express high endogenous RANKL [9] and fail to express functional AR [42]. ARCaP_M_ cells were found to express lower levels of integrin α_2_ than indolent ARCaP_E_ cells (Additional file 4: Figure S3C).

### Restoration of AR expression in LNCaP^RANKL^cells *in vivo*and in 3-D suspension culture enhances cell adhesion to ColI

**Figure 3:**
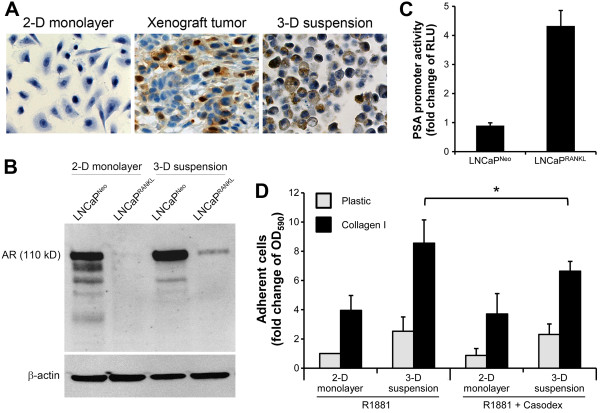
**Restored AR expression in LNCaP** ^**RANKL**^ **cells grown in 3-D suspension increases cell adhesion to ColI. (A)** IHC analysis indicates that the suppressed AR expression in LNCaP^RANKL^ cells could be restored, when the cells were grown as a xenograft tumor or in 3-D suspension culture. Arrows denote AR nuclear localization. **(B)** Restored AR in LNCaP^RANKL^ cells grown in 3-D suspension was confirmed with western blotting. While AR expression is high in LNCaP^Neo^ cell grown in 2-D monolayer or 3-D suspension, LNCaP^RANKL^ cells express AR protein only under 3-D suspension conditions. **(C)** The restored AR is biologically functional since it could promote PSA promoter activity as detected by a PSA promoter-luciferase reporter assay, which showed 4.3-fold increased activity in 3-D suspension culture compared to the LNCaP^RANKL^ cells in 2-D monolayer. Each black bar represents relative light unit (RLU) as a fold difference of 3-D suspension/2-D monolayer for each cell line. **(D)** The function of the restored AR protein in regulating cell adhesion was indicated by treating LNCaP^RANKL^ cells with the synthetic androgen R1881 (10 nM, 48 hours), which resulted in significantly enhanced cell adhesion to ColI. On the other hand, the antagonistic effect of the anti-androgen casodex (20 nM) was seen mainly under 3-D suspension culture conditions. Each value is the mean ± SD of 2 independent experiments done in triplicate.

Since AR is diminished in metastatic LNCaP^RANKL^ cells compared to their parental LNCaP^Neo^ cells, we tested the possibility of restoring AR activity by growing LNCaP^RANKL^ cells in *in vivo* as tumor xenografts or 3-D suspension cultures. As shown in Figure 3A, AR protein expression in LNCaP^RANKL^ cells is undetectable when grown as a 2-D monolayer. IHC staining of AR revealed positive staining of LNCaP^RANKL^ cells grown subcutaneously (Figure 3A). Further, AR IHC staining was also observed in 3-D suspension cultures of LNCaP^RANKL^ cells and this was confirmed by Western blot (Figure 3A, 3B). The restored AR in LNCaP^RANKL^ cells was shown to be biologically functional as revealed by increased PSA promoter luciferase activity in LNCaP^RANKL^ cells (Figure 3C). PSA promoter luciferase activity was significantly elevated in LNCaP^RANKL^ cells grown in 3-D suspension culture as opposed to 2-D monolayer culture. Because AR was shown to drive integrin α_2_ expression in PCa cells [23, 43], we asked if restoration of AR expression in LNCaP^RANKL^ cells, grown in 3-D suspension, enhanced cell adhesion to ColI. LNCaP^RANKL^ cells grown on 2-D monolayer or in 3-D suspension were treated with 10nM R1881, an androgen agonist, or 10 nM R1881 plus an AR antagonist, Casodex (Bicalutamide, 20nM). Cell adhesion to ColI was examined relative to plastic as a control. Under R1881 treatment, in either 2-D monolayer or 3-D suspension culture, LNCaP^RANKL^ cell adhesion to ColI compared to plastic control was significantly higher by 4- and 9-fold, respectively. Casodex was found to block the adhesion of LNCaP^RANKL^ cells to ColI by 1.3-fold in 3-D suspension culture. As expected, AR antagonist did not affect the ColI binding of LNCaP^RANKL^ cells when grown in a 2-D monolayer because of the absence of detectable AR expression under this culture condition (Figure 3D). These results suggest that activated AR, in the presence of R1881 treatment, induces integrin α_2_ expression and is responsible for the increased adhesion of LNCaP^RANKL^ cells to a ColI substratum.

### Restoration of AR expression by downregulating transcription factor AP-4

**Figure 4:**
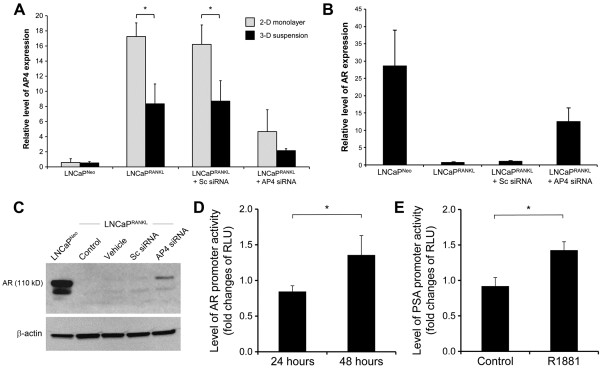
**AR restoration was due to the suppression of AP-4 transcription factor upon 3-D suspension culture. (A)** 3-D suspension culture provided a suppressing condition for the differential AP-4 expression observed in RANKL-overexpressing cells in 2-D monolayer culture. LNCaP^RANKL^ cells transfected with AP-4 siRNA could further reduce AP-4 expression as shown with qRT-PCR analysis. **(B)** AR and AP-4 appeared to have an inverse relationship of expression as detected with qRT-PCR analysis. **(C)** Western blotting analysis was conducted to demonstrate AR restoration upon AP-4 suppression by specific siRNA knockdown. **(D)** The effect of AP-4 appeared to modulate AR transcription, as determined with transient AR promoter reporter luciferase assay. For each condition, RLU was shown as the fold difference of AP-4 siRNA/SC siRNA transfection. **(E)** Effect of AP-4 on AR function was assessed by PSA-promoter reporter luciferase assay. In this experiment, AP-4 was first knocked down in LNCaP^RANKL^ cells. AR responsiveness to androgen R1881 was then measured by PSA promoter reporter luciferase assay.

Our previous publication using site-directed mutagenesis and transcription factor deletion/interference assays identified the suppressive action of AP-4 on AR expression [11]. We hypothesized that decreased AP-4 expression could contribute to increased AR expression in LNCaP^RANKL^ cells grown in 3-D suspension. The qRT-PCR study of AP-4 and AR expression, in LNCaP^RANKL^ cells grown in 3-D suspension or in LNCaP^RANKL^ cells after transient transfection with AP-4 siRNA in 2-D monolayer, revealed an inverse relationship between AP-4 and AR expression. Unlike LNCaP^Neo^ cells, LNCaP^RANKL^ cells grown as 2-D monolayer expressed higher AP-4 with corresponding lower AR. Upon AP-4 knockdown or in cells grown in 3-D suspension, AP-4 expression is reduced and this corresponds with increased AR expression (Figure 4A, B). AR restoration was confirmed by both Western blot and increased AR promoter luciferase activities (Figure 4C, D). In support of the above observations, we also showed that the restored AR was functional, capable of driving increased PSA-promoter luciferase activity by 1.6 fold (Figure 4E). In resemblance to AP-4 which induced EMT in colorectal cancer [44], AP-4 siRNA transfected LNCaP^RANKL^ cells exhibited mesenchymal-to-epithelial transition (MET), a reversal of EMT biomarker expression and decreased cell invasion (Additional file 5: Figure S4A, B). Taken together, AP-4 could be the molecular basis of AR restoration in LNCaP^RANKL^ cells cultured in 3-D suspension. Interestingly, however, enhanced AR expression by gene transfer into LNCaP^RANKL^ cells did not affect AP-4 expression (Additional file 5: Figure S4C), suggesting that there is no established feedback loop between AR and AP-4.

## Discussion

**Figure 5:**
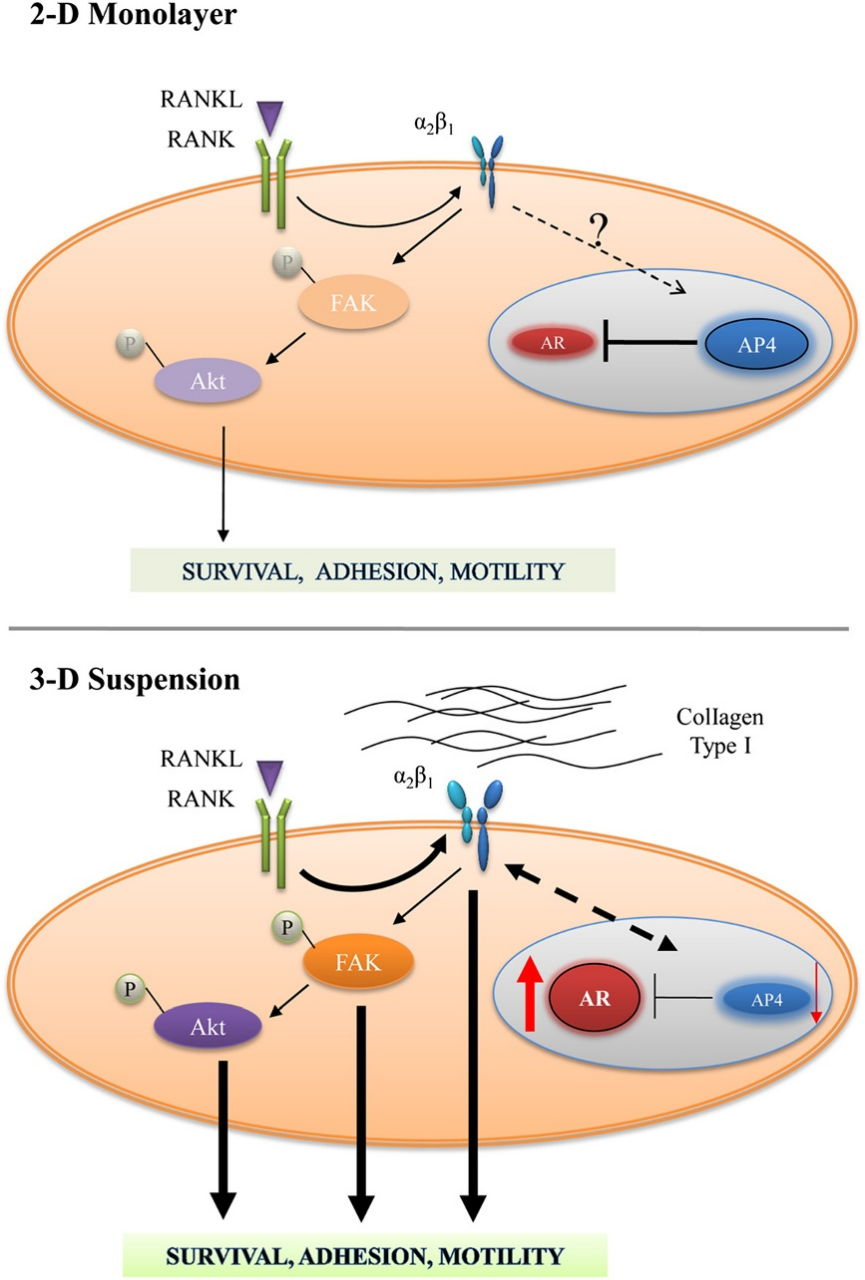
**Schematic summary of the role of RANKL-overexpression in promoting cancer cell adhesion.** In LNCaP^RANKL^ cells, RANKL overexpression induced the expression of α_2_ integrin and AP-4 transcription factor. The later may account for the suppressed AR expression. In 3-D suspension culture, α_2_β_1_ integrin was activated through RANKL expressed by PCa cells or soluble RANKL expressed by stromal cells in the bone microenvironment. This activation would elicit FAK and Akt phosphorylation, resulting in enhanced cell motility, adhesion and survival. α_2_β_1_ integrin activation was further enhanced through AP-4 downregulation, resulting in AR accumulation that could play a role in LNCaP^RANKL^ cell adhesion to ColI. We propose a possible positive feedback loop (dotted line) between AR and integrin α_2_ regulation that is further enhanced under 3-D conditions to support cell anchoring and survival in the bone microenvironment.

The bone environment is enriched with cytokines, growth factors, progenitor cells, and hematopoietic cells, providing a suitable metastatic microenvironment to promote PCa tumor cell adhesion, proliferation, migration, and survival. Despite this supportive microenvironment, cancer bone metastasis is a highly inefficient process and occurs infrequently in cancer patients [45]. However, 80% of all PCa metastatic lesions exist in the bone [4, 5]. To understand the interactions between the tumor and its microenvironment, we engineered an indolent human PCa cell line, LNCaP, with RANKL. We examined LNCaP^RANKL^ and ARCaP_M_ cells, which endogenously expressed a high level of RANKL, for their metastatic potential to bone and soft tissues. The results consistently showed that RANKL drives these cells to undergo EMT and assume many characteristics considered as metastatic cancer cell phenotypes, including the expression of mesenchymal and stem cell biomarkers, neuroendocrine and osteomimetic properties [46], gaining the propensity for metastasis to bone and soft tissues in mice [11]. Further, Chu et al. [11] showed that RANKL protein administered by the intra-peritoneal route can induce prostate cancer bone colonization in mice, confirming the importance of the pathophysiological role of RANKL as both autocrine and paracrine factor. In this study, we specifically examined the effects of the RANKL-RANK mediated signal network that drives PCa cells to express selected integrin isotypes favoring their adhesion to collagens, known to be rich in the bone microenvironment. Our work reveals the importance of the 3-D culture environment that determines integrin expression via functional AR and ultimately affects the pathophysiology of PCa metastases. The pathophysiologic significance of our findings is depicted in Figure 5. 1) RANKL/RANK signaling augments integrin α_2_ expression in RANKL-transfected LNCaP cells but not in ARCaP cells overexpressing RANKL intrinsically (Figure 2, Additional file 4: Figure S3). This observation could possibly be due to the nearly undetectable levels of AR expression in the ARCaP cell line [42]. This suggests that other cell surface receptors, such as discoldin domain receptors [47, 48], glycoprotein VI receptor [49], leukocyte-associated Ig-like receptor [50] or mannose receptor [51, 52], could be downstream targets of the RANK-mediated signal network that controls ARCaP_M_ cell progression and metastasis by interacting with collagen matrices. 2) Consistent with the high bone metastatic behavior of LNCaP^RANKL^ cells, we have shown for the first time that integrin α_2_ expression is significantly enhanced in a 3-D suspension model in a RANKL-dependent manner (Figure 2). Exacerbated integrin α_2_ expression increases the binding of these cells specifically to ColI, the most abundant bone matrix protein (Figure 1). Their profound cell binding to ColI and migration can clearly discriminate indolent LNCaP^Neo^ and metastatic LNCaP^RANKL^ cell lines when cultured in 3-D suspension. Concurrently, we observed that anti-α_2_β_1_ antibody effectively antagonized LNCaP^RANKL^ cell binding to ColI matrix (Figure 1). Enhanced integrin α_2_ expression was shown to facilitate the adhesion and survival of PCa cells through activated FAK and Akt phosphorylation (Figure 2). High expression of integrin α_2_ in metastatic PCa and its important role in cell survival and adhesion in the bone microenvironment is supported by recent experimental and clinical publications [53, 54]. 3) Concomitant with enhanced integrin α_2_ expression, we also observed that LNCaP^RANKL^ cells grown in 3-D suspension exhibited elevated functional AR expression, a result not seen in 2-D monolayer culture (Figure 3). It worth mentioning, while functional assay of AR on LNCaP^RANKL^ cells showed 4.3 fold increases in 3-D suspension/ 2-D monolayer, the fold difference in AR protein level seemed to be higher. However, we would not expect a linear relationship between AR and its responsive promoter-reporter activity due largely to the efficiency of AR and its accessory transcriptional factors binding to the promoters and also the efficiency of the translational machinery of proteins in cells that ultimately determine the promoter reporter activity. Moreover, inhibition of AR nuclear translocation by Casodex treatment reduced LNCaP^RANKL^ cell adhesion to ColI (Figure 3), suggesting that LNCaP^RANKL^ cell adhesion through integrin α_2_ is potentially AR-dependent. Our data are in agreement with those of Nagakawa *et al*. [23] who showed that integrin α_2_ expression and ColI adhesion could be elevated by AR expression in an AR-transfected PCa cell line, DU145. In support of experimental studies, we used the publicly available human prostate cancer genome data listed in TCGA [55], to confirm a direct correlation between mRNA expression of AR and integrin α_2_ (Spearman’s correlation = 0.60, N = 302). Therefore, the adhesion of PCa cells in the bone microenvironment could be enhanced by modulating AR expression and function. While our study and others suggest that AR could regulate integrin α_2_, we were unable to find evidence that integrin α_2_ directly increases AR activity. Our studies of AR promoter did not reveal any binding sites for integrin α_2._ However, further studies are required to finally conclude whether integrin α_2_ could directly or indirectly regulate AR expression. 4) We further illustrated that AR restoration in LNCaP^RANKL^ cells under 3-D suspension condition is at the transcriptional level via downregulation of a key TF repressor, AP-4 (Figure 4). AP-4 overexpression concerts the upregulation of c-Myc/Max in RANKL-overexpressing PCa cells [11] and drives EMT in colorectal cancer [44, 56]. Similarly, downregulation of AP-4 with a concomitant increased expression of AR and integrin α_2_ in PCa cells results in the reversal of EMT and reduced PC invasion (Additional file 5: Figure S4). Because enhanced AR activity was frequently observed in clinically advanced PCa specimens [57, 58], we hypothesize that enhanced AR expression in LNCaP^RANKL^ tumors could enhance the adhesion and survival of LNCaP^RANKL^ cells in mice. In agreement with clinical observations and the role of AP-4 downregulation, our preliminary data showed that LNCaP^RANKL^ cells overexpressing AR did have enhanced growth when inoculated as subcutaneous tumor xenografts in mice (data not shown). Further *in vivo* studies are warranted to determine if AR expression in LNCaP^RANKL^ cells could confer increased α_2_ integrin expression and bone colonization through adhesion of PCa cells to collagen matrix in the skeleton.

Our work reveals the importance of the 3-D culture environment that determines integrin expression via functional AR and ultimately affects the pathophysiology of PCa metastases. Our significant findings are as follows: 1) The ability of PCa cells to adhere, survive and metastasize to bone could be masked by culturing PCa cells as a 2-D monolayer. We observed that RANKL-overexpressing PCa cells have barely detectable AR when cultured on plastic. When these cells were grown as 3-D suspensions or in mice, AR was found to be restored and to activate PSA promoter-luciferase activity. Additionally, we observed higher adhesion of LNCaP^RANKL^ cells to ColI in an AR-dependent manner, most likely through increased expression of α_2_ integrin. These results are consistent with the high levels of AR expression in clinical PCa bone metastasis specimens. 2) The TF repressor, AP-4, was found to be a negative regulator of AR at the transcriptional level and is modulated in a cell context-dependent manner. We speculate that AP-4 downregulation, epigenetically via promoter methylation or genetically via AP-4 regulators such as c-Myc, could play a decisive role in upregulating the levels of AR. This could profoundly control the responses of prostate tumors to androgen deprivation therapy. 3) Upregulation of integrin α_2_ may be a common path for human PCa to develop castration resistance and bone metastasis. Balasubramaniam *et al*. recently studied BAF57, a component of the switching-defective and sucrose nonfermenting (SWI/SNF) chromatin-remodeling complex conglomerate [59]. They found that BAF57 deregulation circumvented androgen-mediated signaling, upregulated α_2_ integrin expression, altered other SWI/SNF complex components at the α_2_ integrin locus and conferred a prometastatic migratory advantage on PCa cells that could contribute to castration resistance and bone metastasis in patients [60]. Hall *et al*. [36] demonstrated experimentally that LNCaP cells selected for ColI binding exhibit higher integrin α_2_ expression, become more adhesive and migratory in *in vitro* and acquire the capacity to grow within the bone compared to non-collagen binding parental cells. These results are in agreement with work of Colombel *et al*. [54] who showed that higher α_2_β_1_ protein expression in primary PCa tissues correlates with bone metastasis. Additionally, Sottnik *et al*. [53] demonstrated elevation of α_2_β_1_ protein level in PCa skeleton metastases when compared to primary site or soft tissue metastases. These data along with our observations (Figure 2) suggest that ColI can re-program cell fates by forcing the expression of α_2_. Ultimately, through a RANKL- and α_2_-mediated downstream signal network ColI can transdifferentiate or reprogram non-metastatic PCa cells to gain increased adhesion, growth and survival potential in the bone microenvironment. More work is needed to build clinically relevant alternative cell signaling network models that could improve cancer diagnosis and prognosis and offer targets for therapeutic intervention.

## Conclusion

The survival of patients with skeletal metastasis is very poor and more efficient prevention therapies are urgently needed. Our understanding of the role of the RANKL/RANK and AR axes in cancer cell adhesion is evolving. Our study supports direct regulation of integrin α_2_ and adhesion to ColI through RANKL/RANK signaling. Previous experimental and clinical studies in agreement with our data show the direct role of AR in ColI adhesion, possibly through integrin α_2_ expression. Our findings suggest that increased integrin activity enhances bone adhesion in a RANKL/RANK and AR dependent manner. Since there are studies supporting the regulation of AR through cell-ECM interaction, it is plausible that a positive feedback loop between AR and integrin α_2_ is induced under 3-D and *in vivo* conditions to support the growth and survival of PCa cells through activated p-FAK and p-Akt. We anticipate expanding the described 3-D suspension culture into co-culture models where relevant cancer cells and normal cells of different lineages can be constructed, studied and fully characterized. We believe that 3-D suspension culture and the co-culture of cancer cells with relevant cells in the tumor microenvironment could provide important additional insights into cancer plasticity and progression.

## Materials and methods

### Cell culture

#### Cell lines and 2D culture conditions

LNCaP human prostate cancer progression models were established by our laboratory as previously described [61]. The LNCaP^Neo^, LNCaP^RANKL^, and LNCaP^RANKL^ cells with RANK knockdown (LNCaP^RANKL-RANK KD^) cell lines were established by Chu *et al*. [11]. LNCaP was maintained in RPMI 1640 supplemented with 5% FBS. LNCaP^Neo^, LNCaP^RANKL^, and LNCaP^RANKL-RANK KD^ cells were also maintained in RPMI 1640 (Invitrogen, Carlsbad, CA) supplemented with 5% FBS with 200 ng/ml of Geneticin selector. ARCaP_E_ and ARCaP_M_ cells established by our laboratory [34, 42] were maintained in T-medium (Invitrogen, Carlsbad, CA) supplemented with 10% FBS. MC3T3 cells (kindly provided by Dr. Neale Weitzmann, Emory University, Atlanta, GA) were maintained in DMEM supplemented by 10% FBS. All cells were incubated in 5% CO_2_ atmosphere at 37°C.

### 3D culture conditions

#### Hydrogel

Hydrogel was prepared using the HyStem Hydrogel kit (Glycosan BioSystem Inc. CA) according to the manufacturer’s instructions. In brief, Hystem and Extralink solutions were prepared by dissolving the lyophilized solids in DG water under aseptic conditions (1% w/v). Extralink was added to the HyStem in 1:4 ratio. The final solution was then incubated for 10 min before encapsulating the cell pellet (10,000 cells/ ml). 200 μl of the final solution was then plated in ultra-low attachment 24-well plates (Sigma) within 20 min of encapsulation for full polymerization in the 37°C incubator for 30 min before adding 1 ml complete medium per well. Collagen type I (BD Biosciences, 100 mg/ml rat tail) was added to the cell pellet at the 0.1 or 0.3 mg/ml final concentration (PH 7.0) before encapsulation, when specified. Medium was changed every 3 days by removing 500 μl/well of the medium and replacing it with 500 μl of fresh complete medium.

#### Suspension

LNCaP^Neo^ and LNCaP^RANKL^ cells, cultured on plastic, were trypsinized at the log phase, washed and resuspended to a final concentration of 20,000 cells/ml. One ml of the final solution was then plated in ultra-low attachment 24-well plates (Sigma). Cells were maintained in RPMI 1640 supplemented with 5% FBS, incubated in 5% CO_2_ atmosphere at 37°C and observed up to 12 days to assess growth and morphology before harvest for additional analysis. For ColI embedded cells, 0.1 or 0.3 mg/ml of ColI was added to the cell pellet before adding the medium. Medium was changed every 3 days by removing 500 μl/well of the medium and replacing it with 500 μl of fresh complete medium.

#### Mesh

Poly (D,L-lactide-*co*-glycolide) (PLGA) fiber sheets (Mesh) were kindly provided by Dr. JurgenGroll, University Hospital, Würzburg, Germany [62]. Sheets 120 μm thick were trimmed to 8 mm circles using a disposable biopsy punch (Kia Medical, Inc) and placed in 48-well plates. Wells were washed with 70% EtOH and twice with 1X PBS. Plates were left under UV light for 30 min before use. LNCaP^Neo^ and LNCaP^RANKL^ cells were trypsinized at the log phase, washed and resuspended to a final concentration of 10,000 cells/ml. 500 μl of the final suspended cells were plated in each well and grown for 7 days before they were fixed or collected for further analysis. To study the ColI interaction with cells, mesh fibers were coated with 50 ng/ul of rat tail ColI (BD Biosciences).

#### Mouse osteoblast matrix (mOBM)

MC3T3-E14, mouse osteoblast precursor cells were grown on 12-well plates (VWR) for 10 days to beyond confluence. Cells were then treated with osteogenic medium (100 nmol/L dexamethasone, 10 mmol/L beta-glycerophosphate, and 0.05 mmol/L L-ascorbic acid-2-phosphate) for an extra 3 weeks with medium changes every 4 days, and then decellularized using 20nM of sterilized ammonium hydroxide (NH_4_OH) for 30 min,and washed extensively prior to seeding the cells [35, 63].

### *In vivo*experiments

All animal procedures were performed according to an approved protocol from the Institutional Animal Care and Use Committee of Cedars-Sinai Medical Center. LN^RANKL^, LN^RANKL-AR^ cells (2×10^6^ cells/100 μl PBS) were inoculated subcutaneously in 4-week-old male nude mice (Taconic, Oxnard, CA). All mice were followed for total of 45 days. Tumor volume was measured every 3 days.

### Microarray data analyses for AR and integrin α_2_ gene signature

To identify potential correlations between AR and integrin α_2_ in human samples, we used a dataset that primarily included adenocarcinoma prostate cancer samples, the Cancer Genome Atlas (TCGA) dataset (n = 336). Expression data for the TCGA dataset was downloaded from the TCGA data portal (http://www.cbioportal.org/public-portal/index.do).

### Cell morphology

Samples were fixed with 3.7% formaldehyde permeabilized with 0.2% Triton X-100 (Sigma) for 30 min and blocked with 5% Bovine Serum Albumin (BSA) (Sigma) for 1 hour. Samples were then washed with phosphate-buffered saline (PBS), pH 7.4 and incubated with 4′-6-diamidino-2-phenylindole (DAPI) (100 ng/ml, Invitrogen) and Alexa Fluor® 488 and 568 Phalloidin (4 μl/ml, Life Technologies) for 1 hr in the dark for nucleus and f-actin cytoskeleton staining, respectively. Phase-contrast images were then captured using Nikon Eclipse Ti (NIKON instruments Inc.). Organoids were placed on coverslip-bottom chambers (Lab-Tek) and fluorescent confocal images were captured using Leica TCS-SP5 Xconfocol microscopy (Leica Microsystems).

### Microscopic live cell imaging and analyses

2×10^4^ cells/ml were seeded on 12-well plates coated with 50 ng/μl or decellularized mOBM wells. A Nikon Eclipse Ti inverted microscope equipped with an automotive x-y-z stage was used for multiposition and perfect focus system time-lapse microscopy. An environmental chamber was used to maintain humidity, 5% CO_2_, and 37°C temperature. FITC and TRITC filters with a shutter control (Lamda SC, Smart Sutter Controller) and a CCD Head camera (Andor Technology) were used for fluorescent imaging. All imaging was performed using a 10× phase contrast (Nikon Plan Fluor Ph1) objective. All the images were also converted to TIF files for analysis of shape and integrated distanced traveled using CellProfiler 2.1.0 (Broad Institute, Boston, MA).

### RNA isolation and quantitative real-time PCR (qRT-PCR)

Total RNA from cells was isolated using an RNeasy Mini Kit (Qiagen, Valencia, CA) according to the manufacturer’s instructions. RNA concentration was quantified using the Nanodrop-2000 (ThermoScientific). Samples with a 260/280 ratio higher than 1.8 were used for subsequent procedures. Complementary DNA (cDNA) was generated from 1 μg of total RNA using M-MLV reverse transcriptase (Promega, Madison, WI), as instructed. 20 ng of cDNA was subjected to PCR analyses using an AB 7500 Fast detection system (Applied Biosystems, Foster City, CA) at 95°C for 10 min and 40 cycles of 95°C for 30 sec, 60°C for 30 sec, and 72°C for 30 sec, followed by a dissociation curve. The sequences of all primers used are listed in Table 1.

**Table 1:** **Primer sequence for qRT-PCR**

**AR**	Forward:	GACCAGATGGCTGTCATTCA
	Reverse:	GGAGCCATCCAAACTCTTGA
**AP-4**	Forward:	GGAGTATTTCATGGTGCCCACT
	Reverse:	GTGGAATGTTGGCAAGGCTAC
**E-Cad**	Forward:	CCACCAAAGTCACGCTGAATA
	Reverse:	GGAGTTGGGAAATGTGAGCAA
**GAPDH**	Forward:	AGCCACATCGCTCAGACA
	Reverse:	GCCCAATACGACCAAATCC
**ITGA2**	Forward:	TGGGGTGCAAACAGACAAGG
	Reverse:	GTAGGTCTGCTGGTTCAG
**Vimentin**	Forward:	GGAAGAGAACTTTGCCGTTGAA
	Reverse:	GTGACGAGCCATTTCCTCCTT

### Western blot analysis

LNCaP^Neo^ and LNCaP^RANKL^ cells were cultured in 6-well plates under 2-D monolayer conditions to 70% confluence or in 3-D suspension conditions for 7 days. The cells were then pelletized and washed with PBS before being lysed in RIPA buffer (1% Triton X-100, 150 mM NaCl, 10 mM Tris/HCl, 1 mM EDTA and 25 mM NaF) containing 1× protease inhibitor cocktail (Roche Diagnostics, Indianapolis, IN). Samples were then centrifuged and the supernatants collected and quantified using the Bradford Protein Assay (Thermo Fisher Scientific, Waltham, MA). 20 μg of cell lysate were resolved on 4-15% Bis-Tris gradient SDS-PAGE (BioRad, Hercules, CA), followed by transblotting onto nitrocellulose membrane (BioRad, Hercules, CA). The membranes were blocked in 5% non-fat milk in TBST for one hour at room temperature (RT) and incubated with diluted primary antibodies in blocking buffer at 4°C overnight. The primary antibodies used were AR (1:500), integrin α_2_(1:500), β-actin (1:2000), AP-4 (1:500, Santa Cruz), FAK (1:1000, Abcam), p-FAK (1:1000), Akt (1:1000), p-Akt (1:2000), c-Met (1:1000), and p-c-Met (1:1000, Cell Signaling). The membranes were washed with TBST three times before incubating with peroxidase-conjugated anti-mouse or anti-rabbit secondary antibodies (1:10000, Santa Cruz) at RT for one hour. After three washes, the membranes were visualized using Kodak Image Station 4000MMProinstrument (AFAB Lab resources, Frederick, MD) and Carestream MI SE Network software. Images were cropped to improve the clarity of the figures. Each image is representative of two separate studies.

### Fluorescence Activated Cell Sorter (FACS)

LNCaP^Neo^ and LNCaP^RANKL^ cells were detached from 2-D monolayer using accutase (Millipore) to preserve membrane receptors. Organoids from 3-D suspension culture were made into single cells using a final concentration of 1 mg/10 ml collagenase in accutase and 20 min incubation at 37°C. Cells were then washed and resuspended into single cell suspension in 1 × PBS containing 1% FBS (FACS buffer). After two washes with cold FACS buffer, cells were incubated for 30 min on ice with FITC-tagged anti-human CD49a, CD49b, CD51/61 and PE anti-human CD29 (BioLegend) or isotype control FITC mouse IgG1, k (eBioscience). Antibodies were washed twice with FACS buffer. Cell fluorescence signals were determined immediately after staining using a BD Accuri C6 flow cytometer (BD Biosciences) equipped with an argon laser emission of 488 nm. FITC and PE were identified using a 530 ± 15 nm and 585 ± 20 nm band pass filter, respectively. The analysis was performed using FlowJo software (TreeStar Inc.). A primary gate was set excluding dead cells or debris based on physical parameters (forward and side light scatter, FSC and SSC, respectively).

### Adhesion assay

The adhesion assay was a modification of a previously published protocol [64]. For each condition, 1×10^5^ cells/ml were placed in 15-ml conical tubes and 10 μg/ml α_2_β_1_ blocking antibody (VLA-2 Millipro) or IgG_1_ isotype control (bioLegend) was added and incubated for 20 minutes at room temperature. Binding assays were performed by seeding 5000 cells in 100 μl of complete medium on plastic or fibronectin-, collagen-IV-, or collagen I-precoated 96-well plates (BD Biosciences). At 30 min, 1-, 3-, 6-, 12- and 24-hr time points, wells were washed twice with PBS, 100 μl of complete medium was replaced, and 10 μlof alamarBlue (Invitrogen) was added, according to the manufacturer’s instructions. After 12 hrs incubation at 37°C in the dark, the plates were read using the Spectra max M2 microplate reader (Molecular Devices, Sunnyvale, CA) at 590 nm with Softmax Pro software. All the readings were normalized to the reading of the well with no cells as a background measurement. The initial activity of the cells was measured by adding 10 μl of alamarBlue directly to the well without washing the cells.

For cells treated with R1881 and/or Casodex, serum-starved medium with 5% dextran-coated charcoal was used instead of complete medium. Plates were washed at 30 min and 1 hr time points and read after 12 hrs of alamaBlue assay. Each condition was performed in triplicate and two independent experiments were carried out per condition.

### Immunohistochemical (IHC) analysis

#### Sample preparation

IHC staining was applied to cells grown as *in vitro 2-D monolayers*. Cells were grown directly on 8-chamber slides to 80% confluence. In some cases, cells were grown as *in vitro 3-D organoids*. Organoids harvested from the 3-D suspension culture were carefully collected into 1.5 ml eppendorf tubes and spun down to a pellet. 1% LMP agarose solution was prepared and added to the pellet. After solidification, using a micro spatula, agarose-cell pellets were wrapped in tissue paper, placed in a plastic tissue cassette, and tissue processing was performed overnight using an automated tissue processor. For *in vivo tissues*, subcutaneous tumors were collected and fixed in 4% formaldehyde immediately for 24 hrs. The next day, tumors were processed for paraffin embedment as described above.

#### Histology analysis

IHC was followed according a previously published protocol [10]. All reagents from the DAKO system (Carpinteria, CA) were used for immunoperoxidase staining of the sectioned slides. Paraffin-embedded slides were rehydrated and antigenic epitopes were retrieved in citrate buffer using a pressure cooker. After antigen retrieval, slides were blocked with dual endogenous enzyme block (DEEB) at RT for 10 min and incubated with primary antibodies against AR (Santa Cruz) at 4°C overnight. The slides were placed at RT for 1 h, rinsed in Tris-buffered saline with 0.05% Tween (TBST) and incubated with Envision + Labeled Polymer-HRP at RT for 30 min. The slides were incubated with peroxidase substrate buffer with a chromogen, diaminobenzidine (DAB), to detect the staining signal, followed by hematoxylin counterstaining of nuclei. After dehydration and cover-slipping, the slides were examined by light microscopy. For monolayer samples, slides were blocked without the peroxidase step.

### Transient transfection and luciferase reporter assays

AR [65] promoter-luciferase plasmid DNA and control CMV-TK plasmid DNA (for transfection efficiency control) were transiently transfected into prostate cancer cells using Lipofectamine 2000 (Invitrogen, Carlsbad, CA) for 48 hrs. The cells were then harvested and protein lysate extracted using 1× passive lysis buffer (Promega, Madison, WI). The lysate was centrifuged at 13,200 rpm at 4°C for 10 min, and the supernatant was collected for luciferase assay. Promoter and TK activity was measured using Dual-Glo luciferase assay, as instructed (Promega, Madison, WI). In short, 20 μl of protein lysate was mixed with 100 μl of substrate (luciferin) and luciferase activity was measured using a BD Monolight 3010 luminometer (BD Pharmingen, San Diego, CA). TK activity was measured by immediately adding 100 μl of Stop&Glo buffer with 50× Stop&Glo substrate to the mix and re-measuring the samples. The relative luciferase activity of each sample was calculated by normalizing to the TK activity. To assess PSA [66] promoter-luciferase activity, we followed the same procedure as above. In addition, cells were serum-starved for 24 hrs before treatment with either 10 nM ethanol or R1881 for another 48 hrs before harvest. Each condition was done in quartet and two independent experiments were carried out per assay.

### Migration and invasion assays

Cell migration and invasion analysis were performed in a 24-well plates using Transwell™ chambers (BD Biosciences). As described previously, transwells were coated with collagen type I or growth factor reduced Matrigel (BD Biosciences) for migration or invasion assay, respectively. LNCaP^Neo^, LNCaP^RANKL-control^ and LNCaP^RANKL-AP-4 KD^ were serum-starved in RPMI 1640 overnight. The next day, transwells were placed on 24-well plates with 400 μl of complete medium. 100 μl of serum-free RPMI 1640 containing 5x10^4^ cells were seeded inside the chambers for 24 hr (migration) or 48 hr (invasion) at 37°C. At each time point, cells remaining on the transwell were fixed with 10% formaldehyde and stained with 0.5% crystal violet. Cells inside the chamber were cleared and remaining cells where quantified [67].

### *In vitro*healing assay and 3D migration assay

24-well plates were coated with 50 ng/μl rat tail ColI and stored at 4°C overnight. Wells were washed twice with PBS before use. For the 2-D wound healing assay, cells were seeded in 24-well plates and cultured to 90% confluence. A straight scratch was made using a 1,000 μl pipette tip to simulate a wound. Wells were washed with 1X PBS to remove unattached cells. Wells were imaged at time zero and after 24 hrs using the 4× objective. Images were analyzed using ImageJ and the distanced traveled was measured. Three images were taken of each triplicate well for two independent experiments. For the 3-D migration assay, organoids were taken from 7-day suspension culture and placed on 24-well ColI-coated plates. Images were captured right after seeding and at 24, 48, and 72 hrs. The area covered by the cells was measured using ImageJ and compared between the two cell lines at each time point. The study was done in triplicate for two independent experiments.

### Cell transfection and transduction protocol

LNCaP^RANKL^ cells were grown in 6-well plates to 60% confluence, then transfected with 100 pmol final concentration of AP-4 siRNA or control siRNA (Santa Cruz Biotechnology, Inc.) for 48 hrs, using Lipofectamine 2000 (Invitrogen, Carlsbad, CA). Samples were collected for qRT-PCR and western blot analysis or further transfected with AR or PSA promoter for the luciferase promoter assay as described above.

For cell transduction, LNCaP^RANKL^ cells were grown in 48-well plates to 50% confluence 24 hrs before transduction. Next day, the complete medium was replaced with complete medium containing Polybrene at a 5 μg/ml final concentration. Cells were infected with AP-4 or control sh-RNA lentiviral particles (Santa Cruz) for 24 hrs. Cells were cultured for an extra 24 hrs before being split to 1:3 ratio. Cell selection was started after an additional 24 hrs with 2 μg/ml of Puromycin.

### Microarray analysis

RNA was isolated as above, hybridized to human U133plus2.0 array, and Affymetrix Gene Chip Expression Analysis was performed (UCLA Clinical Microarray Core). The microarray data was first pre-processed with quantile normalization. Genes were selected based on Students T- tests with P <0.05 and fold changes >2.

### Statistical analysis

Differences between groups were analyzed using Student’s t-test. A p-value < 0.05 was considered statistically significant (denoted by an asterisk). At least three independent *in vitro* experiments were conducted in triplicate for all assays and analyses, unless otherwise specified.

## Electronic supplementary material

Additional file 1: Figure S1: Morphological features of prostate cancer cells in 2-D monolayer and 3-D suspension cultures. The growth of RANKL-overexpressing LNCaP cells was evaluated in 2-D monolayer or in 3-D embedded in hydrogel, on polymeric meshes, and in suspension cultures, in combination with the addition of ColI. The control LNCaP^Neo^ cells formed massive spheroids with hollow lumens (not shown) and exhibited clear invadopodia in the presence of ColI. In comparison, LNCaP^RANKL^ cells formed only loosely-aggregated organoids in 3-D suspension culture, but were mostly in dispersed growth in other cultures. DAPI staining is shown in blue, and F-actin staining is green in 2-D monolayer but yellow in Mesh or 3-D suspension culture. (TIFF 4 MB)

Additional file 2: Table S1: Assessments of 3-D culture conditions. 2-D monolayer culture on plastic was compared with models of 3-D cultures in matrigel, hydrogel, Mesh, and in suspension. 3-D suspension culture was found to be superior in terms of biological relevance, sample production for further molecular analysis, time and cost efficiency, and ease of operation. (TIFF 388 KB)

Additional file 3: Figure S2: Transient differences in the adhesion of LNCaP^Neo^ and LNCaP^RANKL^ cells to ECM proteins. Cells grown on a 2-D monolayer or in 3-D suspension were harvested in single-cell preparation. For each group, 5,000 cells were seeded on 96-well plates coated with ColI, ColIV, or FN. Adhered cells at different times of incubation were determined by alamarBlue assay. Each value is the mean ± SD of 2 independent experiments done in triplicate. (TIFF 333 KB)

Additional file 4: Figure 3: Integrin expression was regulated by RANKL and by the 3-D suspension culture condition. (A) The expression of integrin isoforms was profiled by microarray analysis. Values represented fold changes in LNCaP^RANKL^ cells compared to the LNCaP^Neo^ control. As signified in red, α_2,_ α_v_ and β_3_ integrins had more than 2 fold increases when grown in 3-D suspension. (B) The expression of α_2_ integrin appeared to be dependent on the RANKL/RANK pathway, as reduced expression was seen by qRT-PCR and western blot when the pathway was interfered with RANK knockdown (RANK-KD). (C) Top panels, human prostate cancer ARCaP_E_ and ARCaP_M_ cells grown on a monolayer were stained for α_1_ and α_2_ integrins for FACS analysis. Bottom Panel, quantification of the FACS detection suggested that α_2_ integrin expression was lower in the more aggressive cell line ARCaP_M_ compared with ARCaP_E_ cells. (TIFF 651 KB)

Additional file 5: Figure S4: Suppressing AP-4 led to reversal of EMT and a decrease in cell invasion. (A) LNCaP^RANKL^ cells treated with AP-4 siRNA were studied for EMT markers at the mRNA and protein level. Upon AP-4 KD, vimentin expression was reduced while E-cadherin was increased. (B) LNCaP^RANKL^ cells treated with AP-4 shRNA showed significantly decreased invasive potential, while no changes in migration were observed. (C) AR expression vector was used to express AR in LNCaP^RANKL^ cells (LNCaP^RANKL-AR^). No changes in AP-4 expression were found by qRT-PCR analysis, compared to cells transfected with an empty vector (LNCaP^RANKL-EV^). (TIFF 2 MB)

## Abbreviations

DAPI: 4′-6-diamidino-2-phenylindole
ADT: Androgen deprivation therapy
AR: Androgen receptor
AI: Androgen-insensitive
AS: Androgen-sensitive
BSA: Bovine Serum Albumin
ColI: Collagen type I
ColIV: Collagen type IV
EMT: Epithelial-to-mesenchymal transition
ECM: Extracellular matrix
FN: Fibronectin
FACS: Fluorescence Activated Cell Sorter
FAK: Focal adhesion kinase
IHC: Immunohistochemical
MET: Mesenchymal-to-epithelial transition
mOBM: Mouse osteoblast matrix
PBS: Phosphate-buffered saline
PLGA: Poly (D,L-lactide-*co*-glycolide)
Mesh: PLGA fiber sheets
PCa: Prostate cancer
qRT-PCR: Quantitative real-time PCR
RANKL: Receptor activator of NF kappa-B ligand
3-D: Three-dimensional
TF: Transcription factor.
