# Supplementary figures and images for: Induction of integrin α2 in a highly bone metastatic human prostate cancer cell line: roles of RANKL and AR under three-dimensional suspension culture
Source: Mol Cancer. 2014 Sep 8;13:208. doi: 10.1186/1476-4598-13-208 (PMC4171564; doi:10.1186/1476-4598-13-208)

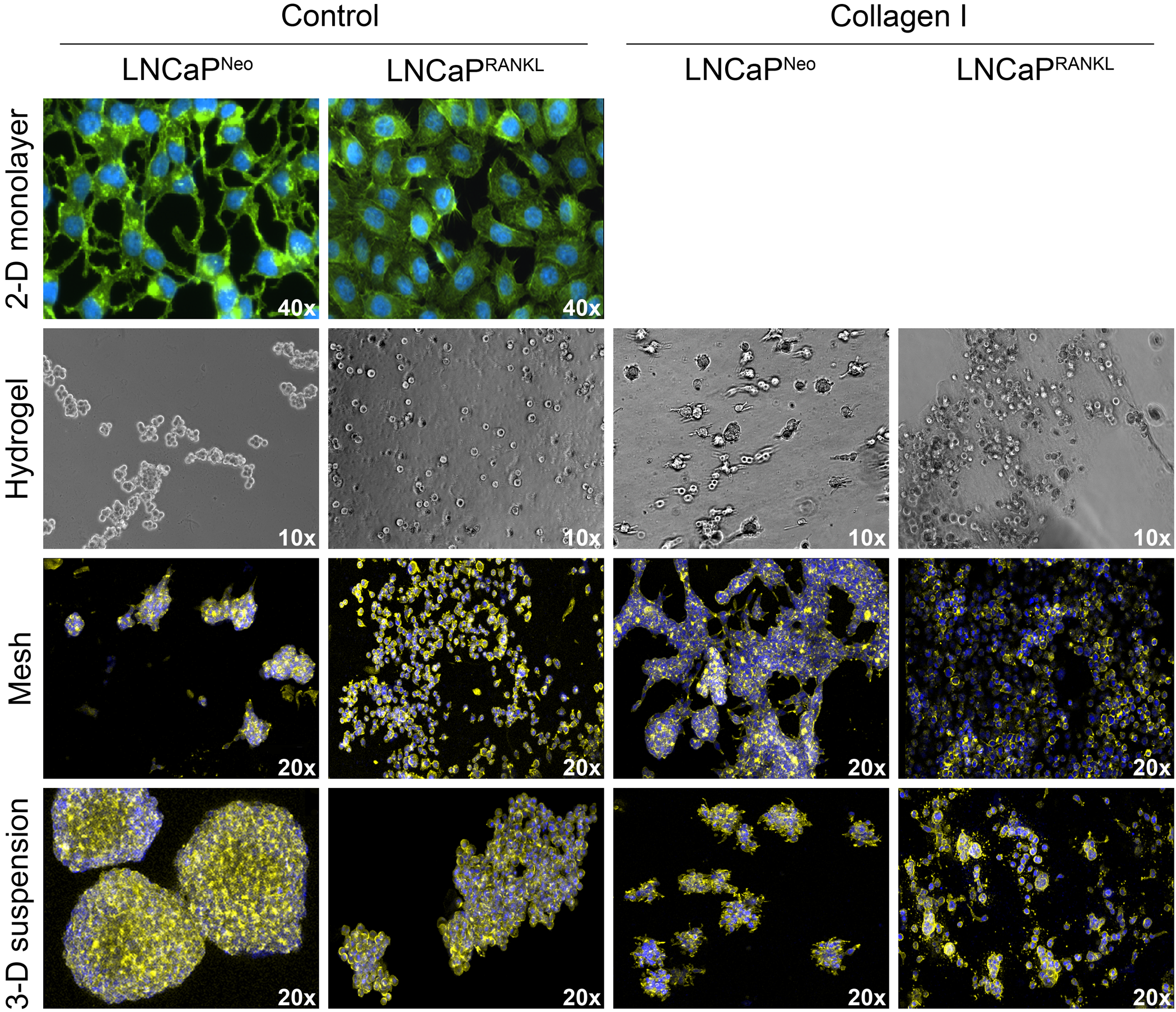

Supplement: Supplementary file 1 — Additional file 1: Figure S1: Morphological features of prostate cancer cells in 2-D monolayer and 3-D suspension cultures. The growth of RANKL-overexpressing LNCaP cells was evaluated in 2-D monolayer or in 3-D embedded in hydrogel, on polymeric meshes, and in suspension cultures, in combination with the addition of ColI. The control LNCaPNeo cells formed massive spheroids with hollow lumens (not shown) and exhibited clear invadopodia in the presence of ColI. In comparison, LNCaPRANKL cells formed only loosely-aggregated organoids in 3-D suspension culture, but were mostly in dispersed growth in other cultures. DAPI staining is shown in blue, and F-actin staining is green in 2-D monolayer but yellow in Mesh or 3-D suspension culture. (TIFF 4 MB) [file 12943_2014_1412_MOESM1_ESM.tiff]

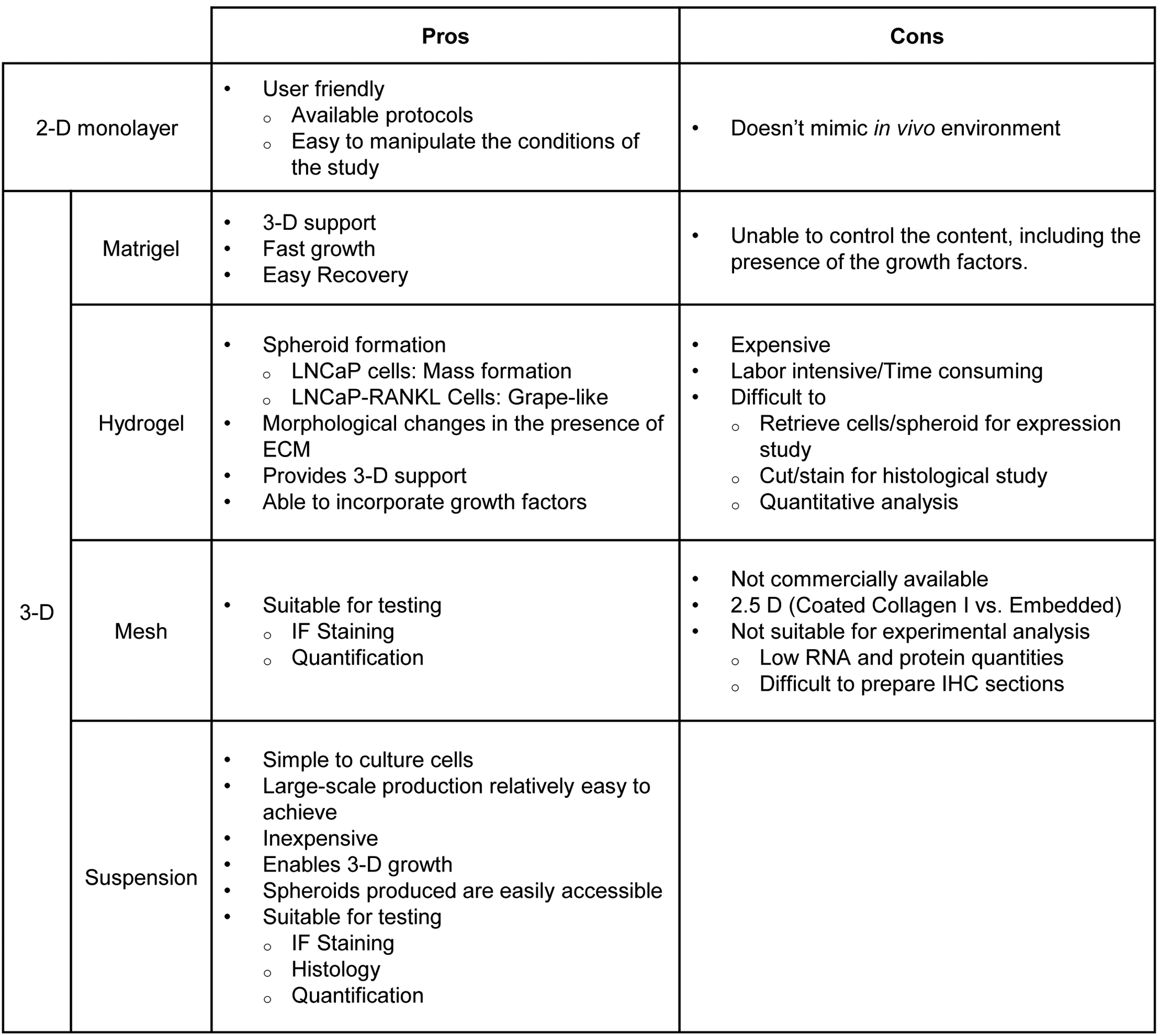

Supplement: Supplementary file 2 — Additional file 2: Table S1: Assessments of 3-D culture conditions. 2-D monolayer culture on plastic was compared with models of 3-D cultures in matrigel, hydrogel, Mesh, and in suspension. 3-D suspension culture was found to be superior in terms of biological relevance, sample production for further molecular analysis, time and cost efficiency, and ease of operation. (TIFF 388 KB) [file 12943_2014_1412_MOESM2_ESM.tiff]

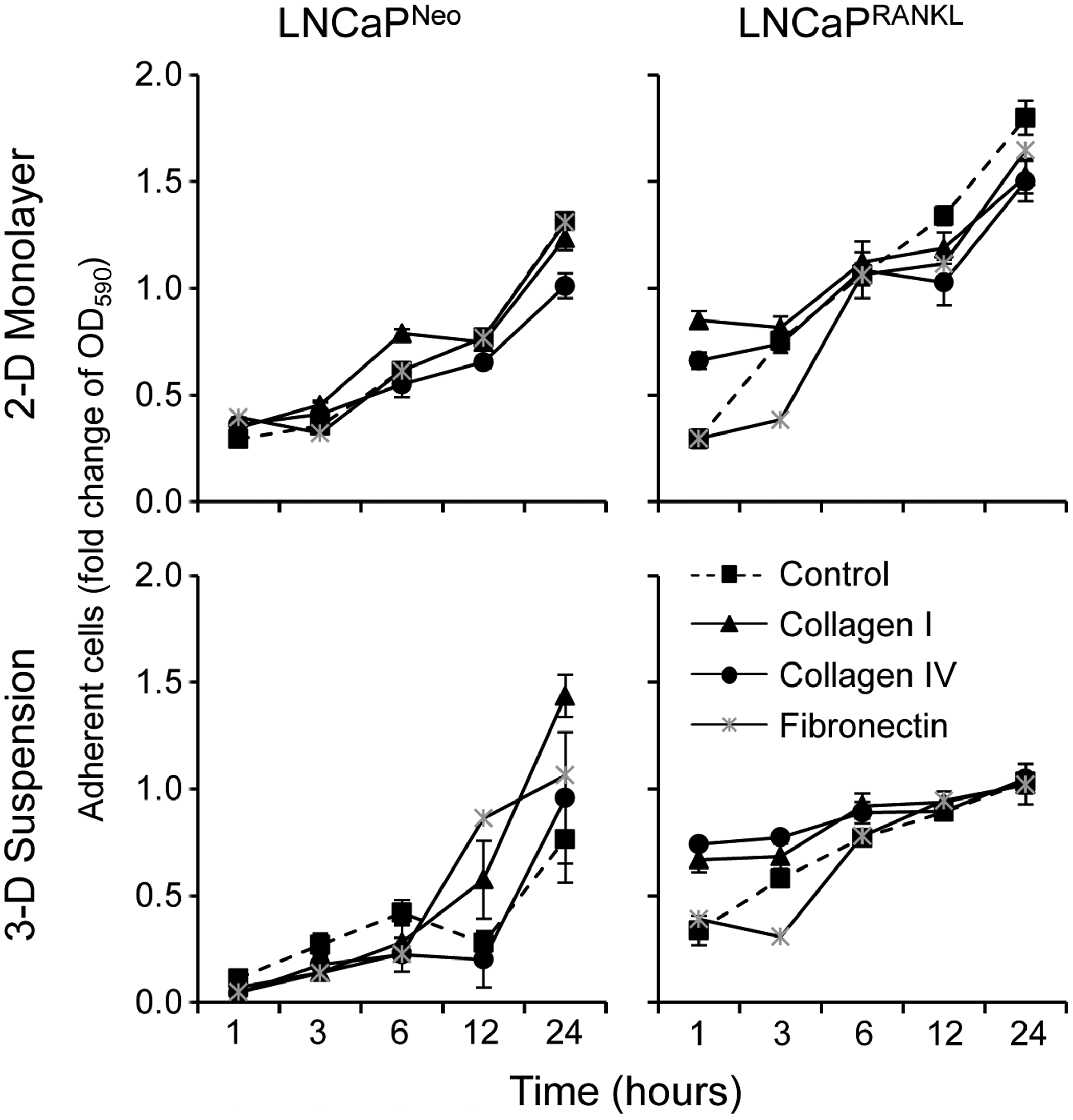

Supplement: Supplementary file 3 — Additional file 3: Figure S2: Transient differences in the adhesion of LNCaPNeo and LNCaPRANKL cells to ECM proteins. Cells grown on a 2-D monolayer or in 3-D suspension were harvested in single-cell preparation. For each group, 5,000 cells were seeded on 96-well plates coated with ColI, ColIV, or FN. Adhered cells at different times of incubation were determined by alamarBlue assay. Each value is the mean ± SD of 2 independent experiments done in triplicate. (TIFF 333 KB) [file 12943_2014_1412_MOESM3_ESM.tiff]

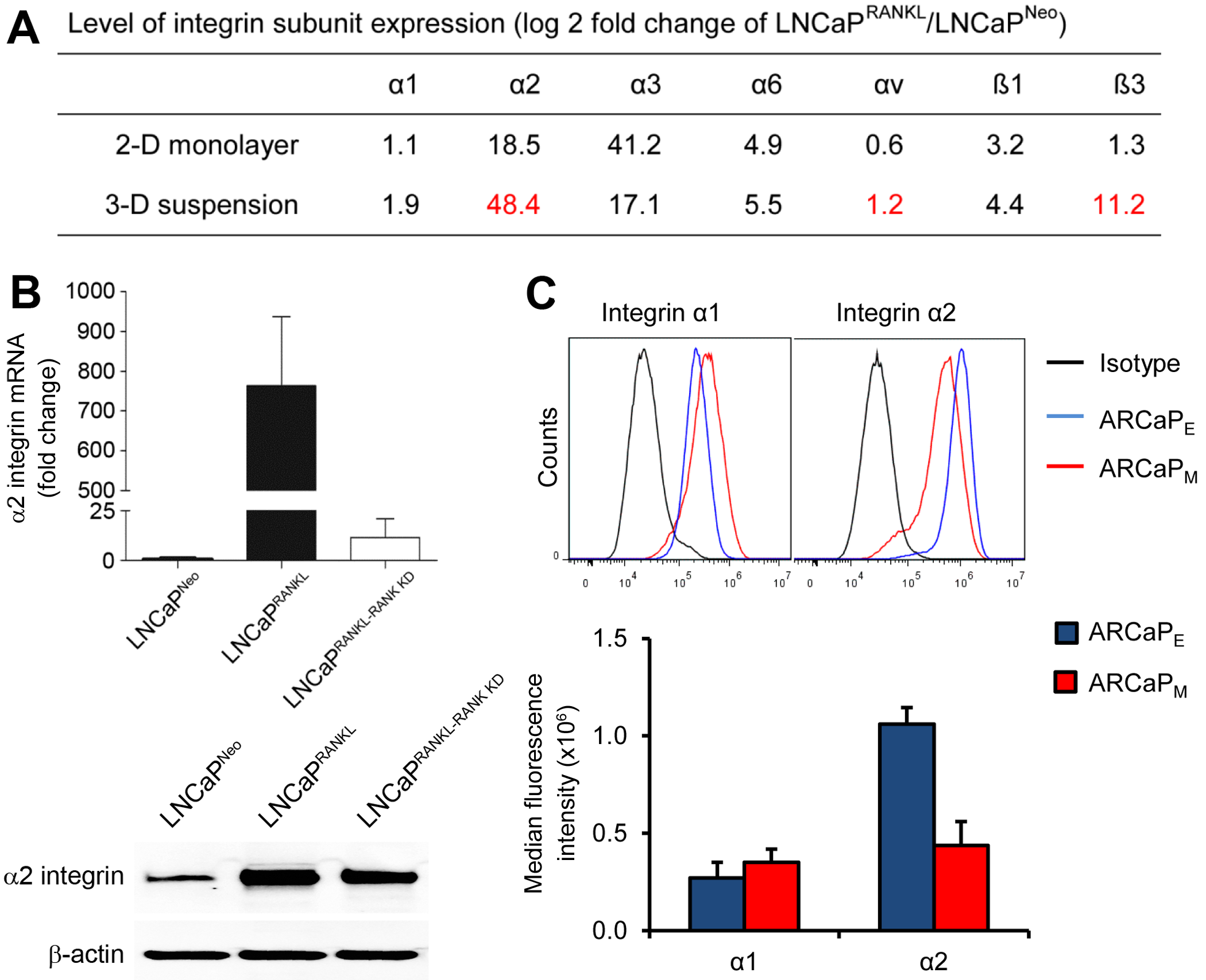

Supplement: Supplementary file 4 — Additional file 4: Figure 3: Integrin expression was regulated by RANKL and by the 3-D suspension culture condition. (A) The expression of integrin isoforms was profiled by microarray analysis. Values represented fold changes in LNCaPRANKL cells compared to the LNCaPNeo control. As signified in red, α2, αv and β3 integrins had more than 2 fold increases when grown in 3-D suspension. (B) The expression of α2 integrin appeared to be dependent on the RANKL/RANK pathway, as reduced expression was seen by qRT-PCR and western blot when the pathway was interfered with RANK knockdown (RANK-KD). (C) Top panels, human prostate cancer ARCaPE and ARCaPM cells grown on a monolayer were stained for α1 and α2 integrins for FACS analysis. Bottom Panel, quantification of the FACS detection suggested that α2 integrin expression was lower in the more aggressive cell line ARCaPM compared with ARCaPE cells. (TIFF 651 KB) [file 12943_2014_1412_MOESM4_ESM.tiff]

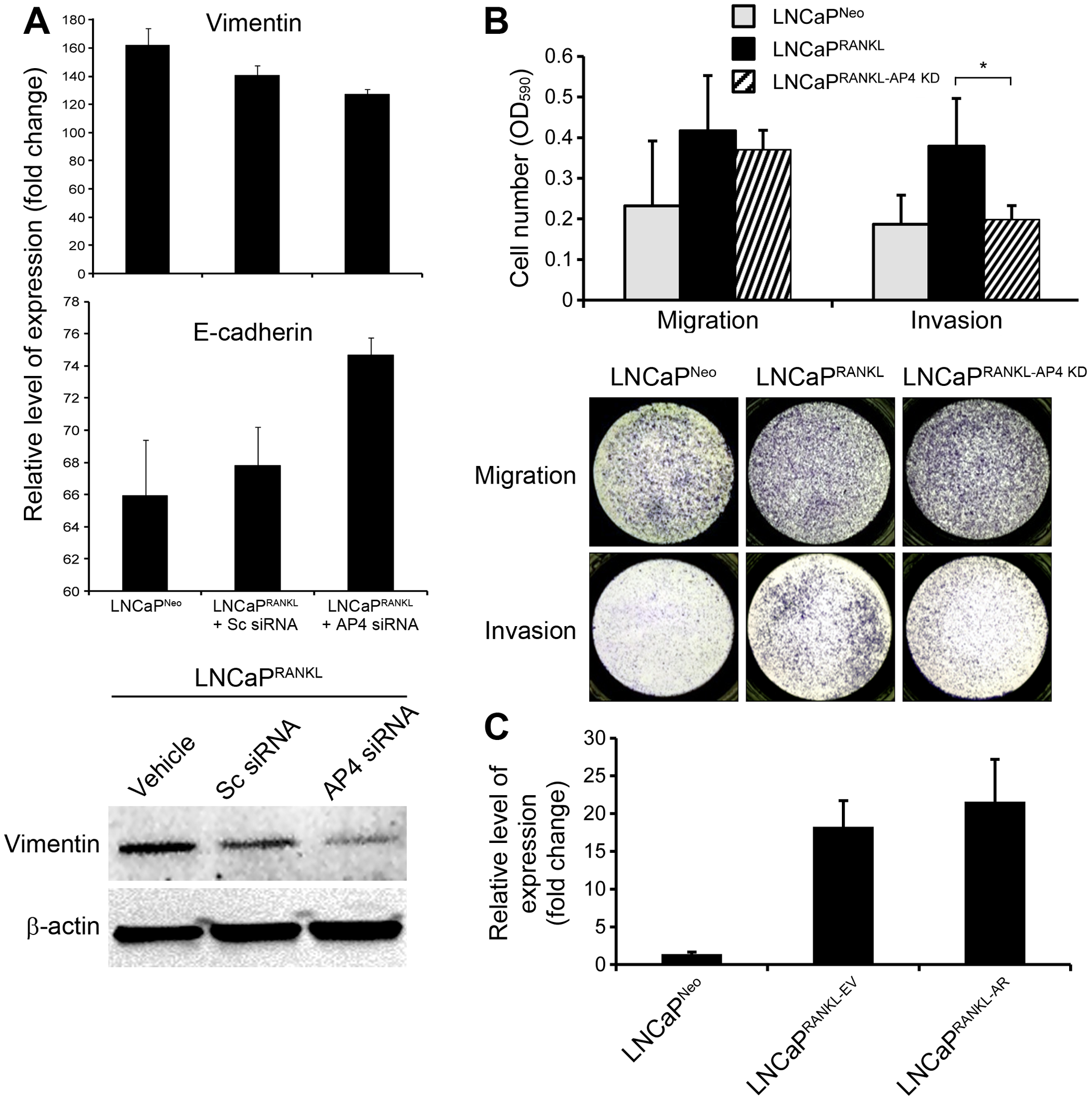

Supplement: Supplementary file 5 — Additional file 5: Figure S4: Suppressing AP-4 led to reversal of EMT and a decrease in cell invasion. (A) LNCaPRANKL cells treated with AP-4 siRNA were studied for EMT markers at the mRNA and protein level. Upon AP-4 KD, vimentin expression was reduced while E-cadherin was increased. (B) LNCaPRANKL cells treated with AP-4 shRNA showed significantly decreased invasive potential, while no changes in migration were observed. (C) AR expression vector was used to express AR in LNCaPRANKL cells (LNCaPRANKL-AR). No changes in AP-4 expression were found by qRT-PCR analysis, compared to cells transfected with an empty vector (LNCaPRANKL-EV). (TIFF 2 MB) [file 12943_2014_1412_MOESM5_ESM.tiff]
